# The Role of Chloride in Raman Signal Enhancement by Electrochemical Silver Oxidation Revealed by Dark Field Microscopy

**DOI:** 10.1021/acs.analchem.4c05942

**Published:** 2025-04-01

**Authors:** Sheila Hernandez, Kevin Wonner, Pouya Hosseini, Paolo Cignoni, Aranzazu Heras, Alvaro Colina, Kristina Tschulik

**Affiliations:** † Chair of Analytical Chemistry II, Faculty of Chemistry and Biochemistry, Ruhr University Bochum, Bochum 44801, Germany; ‡ Department of Chemistry, Universidad de Burgos, Pza. Misael Bañuelos s/n, E-09001 Burgos, Spain; § Max-Planck-Institut für Nachhaltige Materialien GmbH, Max-Planck-Straße 1, 40237 Düsseldorf, Germany

## Abstract

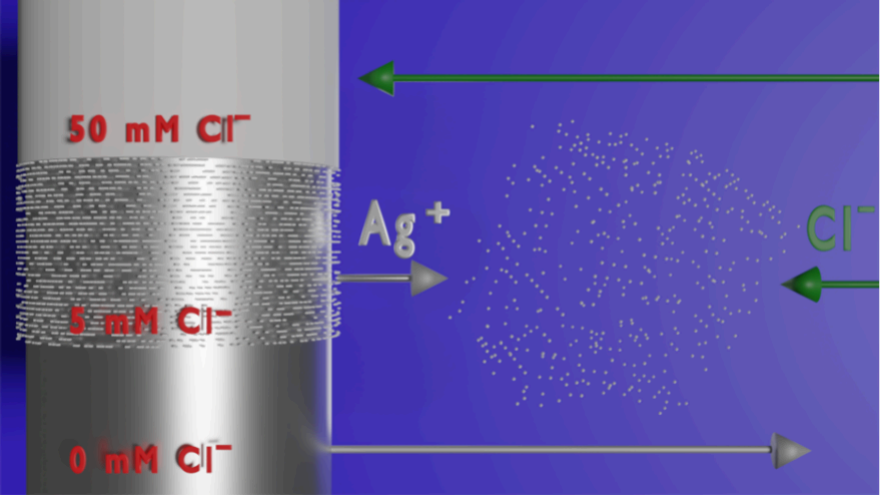

Raman spectroscopy is a widely used technique in several contexts, including chemical analysis, materials characterization, and catalysis. However, to exploit the high capacities of this technique, signal enhancement is needed. For this purpose, several methodologies can be used, and those known as surface enhanced Raman scattering (SERS), or resonance Raman (RR) have been widely used. However, there are some new strategies, such as electrochemical surface oxidation enhanced Raman scattering (EC-SOERS), that require further understanding for optimum exploitation in diverse analytical contexts. In EC-SOERS, the enhancement of the Raman signal is observed during the electrochemical oxidation of silver in the presence of a precipitating agent, but only for specific concentrations of this agent. In this work, we use electrochemical dark-field microscopy (DFM) to explore and reveal the origin of this concentration dependency by monitoring the oxidative formation of EC-SOERS substrates in solutions of different chloride concentrations. These *operando* studies provide a complete picture of the processes taking place on the electrode surface and at the solution adjacent to it with a high time resolution, showing that the formation of the EC-SOERS substrate requires sufficient Cl^–^ to generate AgCl nanocrystals without blocking the surface and allowing the release of Ag^+^ cations. Thanks to the gained mechanistic insights, the selection of a suitable precipitation agent concentration can move from a trial and error selection process to a knowledge-based selection, allowing the rational design of different SOERS substrates that will facilitate the efficient application of SOERS in different research contexts

## Introduction

Raman spectroscopy is a powerful analytical technique that provides information related to the vibrational modes of molecules. This vibrational information makes Raman a highly selective technique, as it can be interpreted as a fingerprint of the studied molecules.^1,2^ The main drawback that has limited its great potential for chemical analysis has been the low intensity of the Raman signal, which results in the poor sensitivity of this technique.

Different strategies to enhance the Raman scattering of molecules can be found in literature.^1,3−5^ The most studied one is surface enhanced Raman scattering (SERS), which was first observed by Fleischmann et al.^6^ in 1974 and further explained by Van Duyne and Jeanmaire,^7^ and Albrecht and Creighton^8^ in 1977. The SERS effect overcomes the classic low-sensitivity limitation of Raman and demonstrates a high amplification of the Raman signal due to the interaction between molecules and a nanostructured substrate, usually with plasmonic properties, allowing the detection of even a single molecule.^9,10^

After intense and in-depth studies of the SERS phenomenon, it was found that two different mechanisms contribute to the SERS effect.^1,11,12^ The first and most significant is the electromagnetic mechanism (EM) due to an increase in the scattered light by resonance with the surface plasmon (SPR, surface plasmon resonance). The second one is the chemical mechanism (CE), which is less efficient but is also required. This CE effect is mainly due to the interaction between the target molecule and the nanostructured substrate due to charge transfer between them, which modifies the energy levels on the “molecule–substrate” complex.

Traditionally, metallic nanomaterials have been the most employed substrates, mainly gold, silver, and copper nanostructures, since their plasmonic band lies in the visible range of the electromagnetic spectrum, leading to unique optical properties. In recent years, new nanomaterials have been developed to enhance the Raman signal. The most notorious are the semiconductors/dielectric nanocrystals.^4,13−15^ In these kind of materials, the mechanism of Raman signal enhancement is quite different; thus, the main mechanism that explains this enhancement is the CE due to the interaction between HOMO and/or LUMO levels of the molecule and the valence (VB) and/or conduction (CB) bands of the semiconductor.^4,16,17^ The contribution of the EM to the Raman signal enhancement in semiconductors/dielectrics is rather discrete, with optical resonances^4,18^ or plasmon resonance in the UV region for the VB and in the IR region for the CB.^15−17^ However, the energies of the VB and the CB can be tuned by modifying the crystal properties, including defects such as vacancies or atoms.

Recently, a methodology was developed to obtain Raman signal enhancement during the oxidation of a silver electrode under specific electrolytic conditions.^19^ This new phenomenon was first observed in 2018 and was denoted as electrochemical-surface oxidation enhanced Raman scattering (EC-SOERS). During this short period since its discovery, SOERS has already demonstrated excellent capabilities in detecting and quantifying different molecules with high reproducibility.^20−22^ This methodology can even be used to analyze complex samples, which makes it a promising methodology for chemical analysis. However, the sample composition (e.g., a high chloride concentration) can influence the Raman response. This can be avoided by a simple pretreatment of the sample^20^ but at the same time suggests that further studies are worthwhile to better understand the effect of conditions on the process. The Raman signal enhancement by this effect is related to the optical properties of the semiconductor/dielectric nanocrystals generated on the surface of the electrode, and the interaction of molecules with metal cations adsorbed on these nanocrystals, as was recently reported.^23^ Therefore, it can be inferred that both EM and CE are involved in this phenomenon, with the charge transfer among molecules, metal cations, and nanocrystals playing a key role.^23^ However, the requirement of specific electrolytic conditions, i.e., a low concentration of a precipitating agent, remains unclear. Initial studies addressing this question show that Cl^–^ concentration modulation also allows switching between SERS and SOERS responses.^21,24^ Conversely, the effect of modulating the precipitating agent on the electrogenerated structures has not yet been investigated. Hence, the role of the precipitating agent during the evolution of the SOERS active substrates and the resulting Raman enhancement remain largely unclear. In this work, we will decipher the origin of this observation by revealing the role of chloride in the formation of SOERS active surfaces and provide experimental evidence of the role of chloride in the generation of SOERS substrates.

This is achieved by coupling dark-field microscopy (DFM) with electrochemistry to study the electrogeneration of EC-SOERS substrates, since the generation of the SOERS substrate can be observed in situ with a high spatial resolution during the electrochemical experiment. The main advantage of this *operando* experiment is that it provides dynamic information about the processes taking place on the working electrode surface and at the solution adjacent to it. That is, it allows us to monitor with high time-resolution and localize with high precision the formation of SOERS active structures, providing valuable information about how, when, and why they are formed.

DFM is a widely used technique in various fields, such as biology and medicine. Recently, some works have been published showing its great potential in studying nanoparticles and nanomaterials.^25−27^ The coupling of DFM with electrochemistry allows the study of redox processes of nanoparticles.^27−29^ Spectral analysis of single nanoparticles is usually made with a spectrometer coupled with DFM, monitoring single particles using hyperspectral images.^25,27−30^ An alternative to track the behavior of plasmonic nanoparticles is to use colorimetric analysis of DFM images.^31−33^ Since the spectroscopic response of plasmonic nanoparticles is accompanied by color changes, the analysis of these spectral changes related to the evolution of RGB intensities and RGB percentage has been proposed.^31−33^ This analysis has been demonstrated to be very useful, providing information about the spectral changes in nanoparticles in a simple way.

DFM has been used extensively to analyze plasmonic nanoparticles. Besides, some works can be found about the study of the generation of silver nanoparticles in solution from a sacrificial silver microwire using DFM,^34,35^ demonstrating the suitability of DFM to study these systems. This work is focused on studying the processes taking place during the silver oxidation with the purpose of shedding more light on EC-SOERS substrate generation. Different amounts of chloride were used in this study in order to investigate the role of the Cl^–^ concentration. In DFM, the sample must be optically transparent, which is not possible in the case of the surface of a conventional silver electrode. In this work, an alternative is proposed, investigating the oxidation products of a 25 μm silver wire. Since Raman signal enhancement in EC-SOERS is highly dependent on the electrolytic conditions,^24^ different electrolyte concentrations are analyzed to understand the different behavior of the Raman response.

## Experimental Section

### Reagents and Materials

Potassium chloride (KCl, ≥99%, Sigma-Aldrich), perchloric acid (HClO_4_, 60%, reagent, Sigma-Aldrich), uric acid (≥99%, reagent, Sigma-Aldrich), and silver nitrate (AgNO_3_, 99.99%, Alfa Aesar) were used. All solutions were prepared by using UV-treated ultrapure water (Millipore) obtained from a Barnstead GenPure xCAD Plus purification system provided by Thermo Scientific (0.055 μS·cm^–1^ conductivity at 23 °C).

### Instrumentation

#### Electrochemical Cell

A customized handmade cell was designed to perform both Raman or DFM coupled to electrochemistry. The cell was made using a glass microscope slide, in which three microwires were attached as electrodes (a 25 μm diameter Ag wire as the working electrode, a 25 μm diameter Pt wire as the counter electrode, and a 15 μm diameter PtIr wire as the reference electrode). A glass coverslip was placed on the top of the cell to prevent water evaporation.^27^ Further details can be found in Figure S1 in the SI.

#### Dark-Field Microscopy (DFM)

DFM images were collected using an Olympus BX43 Microscope (Olympus Deutschland GmbH), using an air 40× objective, linked to a quartz halogen lamp (Fiber-Lite DC950 Illuminator Dolan-Jenner Industries Inc.) and an optical illumination unit (CytoViva Inc.) to focus the light on the sample. Immersion oil of high refractive index (Cargille, type A, refractive index at 23 °C of the D line of 1.5150) was placed between the condenser and the sample to ensure good contact. An optical CCD camera (color Retiga R_1_ OEM Camera) was used to register the scattered light and to obtain the optical images. To perform the spectroelectrochemical experiment, a potentiostat (PalmSens 4) was coupled to the microscope system, and optical images were recorded at 4 frames per second.

#### Raman Spectroscopy

Raman spectra were collected using a Raman Microscope (Alpha-300R confocal Raman microscope, WITec, GmbH) with a laser source of 633 nm. The laser power was 1 mW, and spectra were collected using an air 20× objective (Zeiss EC Epiplan HD 20x/0.4) and 1 s integration time. To perform the spectroelectrochemical experiments, a PalmSens 4 potentiostat was coupled to the microscope system.

#### Scanning Transmission Electron Microscopy (STEM)

STEM images of particles were recorded on a JEOL JEM-2800 electron microscope equipped with a Schottky electron gun working at a 200 kV accelerating voltage, resulting in a point-to-point resolution of 0.14 nm. A carbon film on a 200-mesh gold TEM grid was used (PLANO S-160A, Plano GmbH). The TEM grid was placed below the wire during the experiment to collect particles formed near the wire. After the experiment, the sample was thoroughly rinsed with ultrapure water (Millipore) and dried under a nitrogen atmosphere.

#### X-ray Photoelectron Spectroscopy (XPS)

XPS measurements were carried out in an ultrahigh-vacuum (UHV) setup equipped with a polychromatic Al Kα X-ray source (1486.6 eV; anode operating at 14 kV and 14 mA) and a hemispherical analyzer (type CLAM2, VG, Scientific, Thermo Fischer Scientific). The base pressure in the measurement chamber was maintained at about 1 × 10^–9^ mbar. All spectra were recorded with a pass energy of 100 eV. The sample was prepared by replacing the glass slide holder of the electrochemical cell with a conductive ITO electrode to confirm the particle generation with DFM (data not shown) and to collect the particles landing on the ITO slide. After the experiment, the sample was thoroughly rinsed with ultrapure water (Millipore) and dried under a nitrogen atmosphere.

## Results and Discussion

### Observing EC-SOERS Response from Ag Microwire

EC-SOERS has been reported with different Ag electrodes (screen-printed electrodes, Ag disk electrodes); however, microelectrodes have not been used for this purpose so far. Therefore, the first step will be to confirm that this enhancement is also possible using a Ag microwire as electrode. For that, Raman spectroelectrochemistry (Raman-SEC) experiments were performed using the electrochemical cell described in the Experimental Section and Figure S1 in the Supporting Information (SI). The experimental conditions selected to perform the oxidation of the microelectrode were adopted from previous EC-SOERS studies on macroelectrodes.^19,24^ Linear sweep voltammetry (LSV) was performed in a 0.1 M HClO_4_ and 5 mM KCl solution in the presence of the analyte while simultaneously monitoring the Raman response. The potential-dependent evolution of the Raman peaks will be denoted as VoltaRamangram in order to simplify this terminology.^36^ In this case, 0.1 mM uric acid was selected as test molecule, since this molecule has been previously studied by EC-SOERS, showing a good SOERS response.^19,20^ The potential was scanned from −0.45 (OCP) to −0.05 V at 0.02 V·s^–1^.

**Figure 1:**
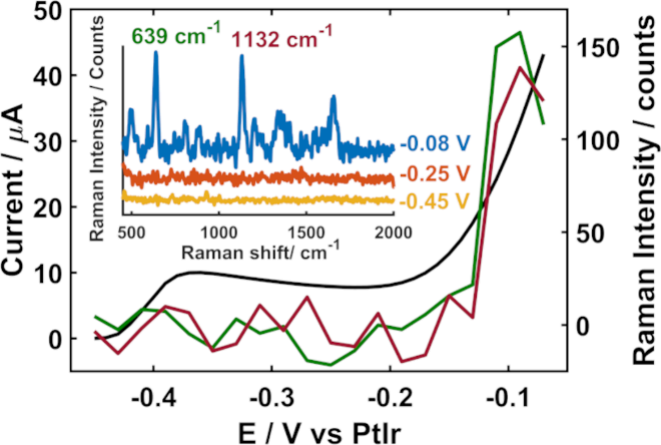
LSV response (black line, left axis) and VoltaRamangrams (right axis) at 639 (green line) and at 1132 cm^–1^ (garnet line) of 0.1 mM uric acid in 0.1 M HClO_4_ and 5 mM KCl as the supporting electrolyte using a Ag microwire as the WE and silver source. The potential was scanned from −0.45 (OCP) to −0.05 V at 0.02 V·s^–1^. The inset shows the Raman spectra for uric acid at −0.45 (yellow line), −0.25 (orange line), and −0.08 V (blue line).

Figure 1 shows the electrochemical response (LSV, black line, left axis) compared with the potential-dependent evolution of two characteristic Raman peaks for uric acid at 639 and 1132 cm^–1^ (VoltaRamangrams, garnet and green lines, respectively, right axis).^19,37^ The Raman spectra, registered at −0.45, −0.25, and −0.08 V vs PtIr quasi-reference electrode, are shown in the inset of Figure 1. The LSV shows an anodic peak at around −0.38 V related to the oxidation of silver to generate AgCl, followed by a further oxidation current, from −0.15 V onward, related to the generation of Ag^+^ ions.

From this figure, it can be inferred that the Raman signal enhancement (see VoltaRamangrams) starts simultaneously with the oxidation of silver to silver ions (see the LSV), as was described in previous works.^19,24^ Note that this is the first demonstration of EC-SOERS on microwires, which not only opens up new gates in the field of microsensors but also allows us to elucidate the role of chloride using microscopic techniques, such as DFM.

### Ag Microwire Oxidation at Different Cl^–^ Concentrations

The same electrochemical cell design and electrochemical conditions were employed in DFM. With the aim of monitoring the changes on the Ag wire during the electrochemical oxidation, the experiment was recorded using a CCD Camera (Video S1).

**Figure 2:**
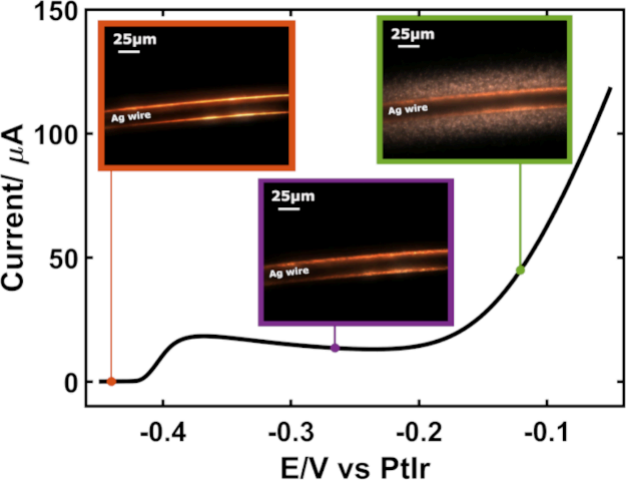
LSV at a Ag microwire in 0.1 M HClO_4_ and 5 mM KCl (black line) from −0.45 (OCP) to −0.05 V vs PtIr. Scan rate: 0.02 V·s^–1^. Representative DFM images recorded at −0.44 (orange frame), −0.27 (purple frame), and −0.12 V (green frame) are displayed.

At the beginning of the LSV (at −0.44 V), the Ag microwire looks bright due to the scattering of light (orange frame in Figure 2). After the anodic peak at −0.38 V (see LSV in Figure 2), which is related to the silver oxidation to generate AgCl (Ag + Cl^–^ → AgCl + e^–^), the edges of the wire show a decrease in brightness (purple frame Figure 2) because of the generation of a nonuniform layer of AgCl particles.^19,24^ When the current starts to grow after −0.20 V due to oxidation of silver to silver ions (Ag→Ag^+^+ e^–^), a high number of bright particles emerges in front of the Ag wire (green frame in Figure 2).

This is related to the generation of Ag^+^ ions, which diffuse away from the electrode surface and form AgCl particles directly in solution (Ag^+^ + Cl^–^ → AgCl). The observed electrochemical behaviors are well-described in literature and can be summarized as follows: the more positive the applied potential, the higher the Ag^+^ concentration near the surface, which is proportional to the enhancement of the Raman signal.^38−44^ When the product of both concentrations ([Ag^+^]·[Cl^–^]) is greater than the solubility product (*K*_sp_ = 10^–9.7^ mol^2^·L^–2^ for AgCl),^45^ AgCl precipitation takes place, generating a nonuniform thin layer of AgCl particles on the surface of the electrode^19,24^ and consuming the chloride present near the wire. From this point onward, in the positive scan direction, more Ag^+^ is generated on the surface. Part of these Ag^+^ cations are adsorbed on the AgCl particles (leading to the enhancement of the Raman signal^23^), while the rest of the Ag^+^ cations can pass through the layer of AgCl particles via spaces between AgCl particles.^38,40^ In the absence of chloride ions (which are depleted by the generation of the AgCl particles next to the Ag wire), Ag^+^ cations can diffuse away from the surface, encountering Cl^–^ from the bulk of the solution and generating the detected bright AgCl particles in solution.

**Figure 3:**
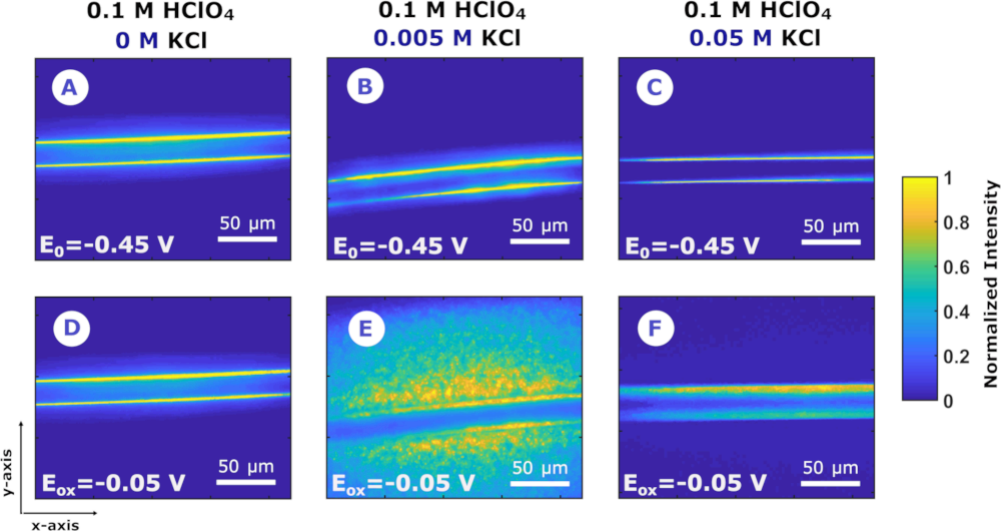
Normalized intensity of the R component of DFM images during LSV at a Ag microwire at (A–C) the initial potential (*E*_0_ = −0.45 V) and (D–F) the oxidation vertex potential (*E*_ox_= −0.05 V) in different aqueous electrolytes: (A, D) 0.1 M HClO_4_, (B, E) 0.1 M HClO_4_ + 5 mM KCl, and (C, F) 0.1 M HClO_4_ + 50 mM KCl.

**Figure 4:**
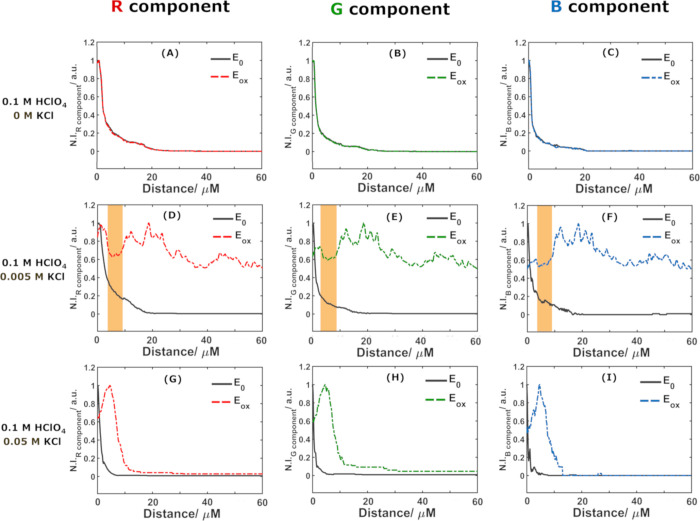
Normalized scattered intensity (N.I.) vs distance from the electrode for the (A, D, G) R component, (B, E, H) G component, and (C, F, I) B component of the CCD signal in 0.1 M HClO_4_ and 3 different chloride concentrations: (A–C) 0 M KCl, (D–F) 5 mM KCl, and (G–I) 50 mM KCl at two different potentials: *E*_0_= −0.45 V (black solid lines) and *E*_ox_= −0.05 V (colored dashed lines).

To validate this suggested mechanism and obtain a better understanding of the process, additional experiments were performed for different chloride concentrations under otherwise identical conditions. These results are shown in Figure 3 as the normalized intensity of the R component of the DFM images registered at two different potentials. Analysis of the optical DFM videos provides a dynamic picture of the generation and growth of AgCl, as well as the deep changes of the wire surface, in these three sets of experiments at different KCl concentration. It should be mentioned that as Ag^+^ is colorless and AgCl is white, the different RGB channels should provide similar results (as seen in Figure 4); therefore, only the R component will be represented. Hence, Figure 3 shows the normalized intensity of the R component in RGB images obtained from the DFM video at −0.45 and −0.05 V (initial and final potential, respectively).

**Figure 5:**
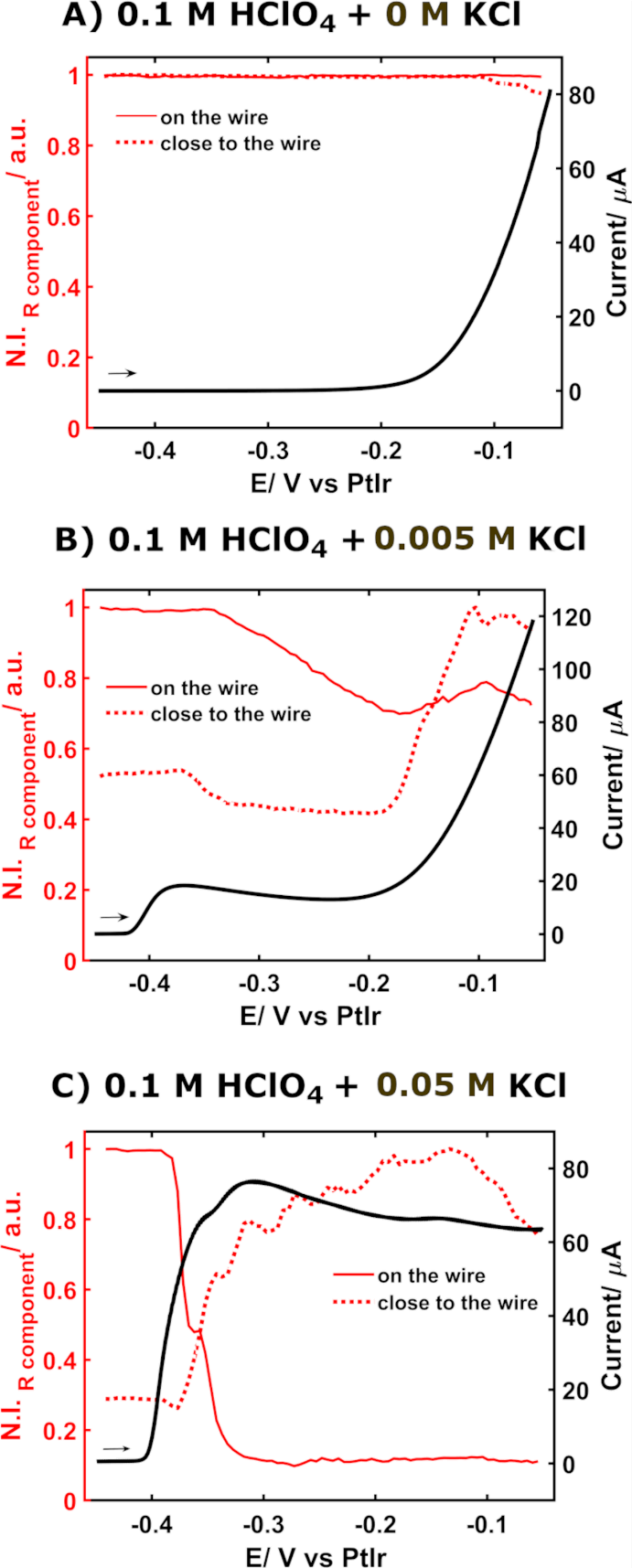
Evolution of the normalized R component intensity (red lines, left axis) with the applied potential at two different positions, on the wire (solid red line) and close to the wire (∼3 μm distance, dotted red line), compared with the electrochemical response (LSV, black line, right axis) during the oxidation of a Ag wire in 0.1 M HClO_4_ with (A) the absence of Cl^–^, (B) 5 mM Cl^–^, or (C) 50 mM Cl^–^.

In the absence of chloride, that is, in high-purity 0.1 M HClO_4_ solution, the wire did not visibly change throughout the oxidation process (Figure 3A and D), although the electrochemical current response related to the oxidation of Ag to Ag^+^ was similar to the previous conditions (0.1 M HClO_4_ + 5 mM, see Figure 5A and B). This is attributable to the fact that Ag^+^(aq) ions are generated during electrochemical Ag oxidation instead of AgCl(s). Since the Ag^+^ ions do not scatter the incident light,^28^ no changes were observed by DFM; at the same time, no changes were noticed on the wire, indicating that the oxidation of Ag does not lead to a significant change in the surface of the electrode.

On the contrary, the higher the Cl^–^ concentration, the more the intensity of the light scattered by the wire will be detected. First, with a smaller concentration of Cl^–^ (5 mM, Figure 3B and E), slight changes can be observed at the edges of the wire, while the most significant change is observed due to the generation of the AgCl particles, which emerge from the wire into the adjacent electrolyte solution.

Finally, the chloride concentration was increased to 50 mM (Figure 3C and F), while the proton concentration was preserved (0.1 M HClO_4_). This experiment shows the most significant optical changes on the wire, leading to a less uniform surface, which is most likely associated with the growth of a much thicker AgCl(s) layer (Figure 3F) compared to that obtained at a chloride concentration of 5 mM. Due to the higher concentration of chloride, much more AgCl is generated on the electrode surface, consuming a much higher amount of Ag^+^ ions and preventing the diffusion of these ions into the solution, due to the growth of this thick AgCl layer.^38^ At the same time, it should be expected that the presence of a thicker layer on the electrode slows the surface oxidation.

In short, the absence of emerging particles is evident in both chloride-free and 50 mM Cl^–^ solutions. This implies that in order to get SOERS enhancement the chloride concentration needs to be low enough to enable the release of Ag^+^(aq) cations from the Ag wire, which then form AgCl particles in the solution. At higher chloride levels, a thick, protective AgCl(s) layer is formed at the wire, preventing the formation of AgCl nanoparticles in solution. The dynamics of the particle formation can also be studied with a further analysis of the DFM videos, which can be displayed in two different ways: (1) by depicting the variation of the intensity along the space during the experiment (Figure 4) or (2) by plotting the variation of intensity in a particular position with the applied potential (Figure 5).

Changes in intensity along the direction normal to the Ag wire (*y*-axis in Figure 3) provide an estimate for the width of the generated AgCl layer on the wire (Figure 3E and F) as well as for the diffusion layer of the AgCl NPs formed in solution (Figure 3E), as seen in Figure 4. Further details on the associated data treatment can be found in Figure S2-A in the SI.

Figure 4 shows the evolution of normalized intensity at the initial (black line) and vertex oxidation potential (color lines: red for R, green for G, and blue for B components in the RGB images of the DFM video). As stated above, only negligible differences in intensity can be found for the different components.

In the absence of chloride (Figure 4A–C), the appearance of the wire and all intensities remain constant throughout the experiment. In contrast, in the presence of 5 mM KCl (Figure 4D–F) a thin layer of particles is observed due to the release of silver ions from the wire, which react with chloride ions present in the electrolyte to form that noncontinuous AgCl layer within the first 5 μm.^38^ Accordingly, chloride ions are consumed near the electrode when AgCl is formed, and at sufficiently positive potentials the rate of Ag^+^ formation exceeds the diffusional flux of chloride from the solution. Hence, a region is formed in which the chloride concentration is too low to continue AgCl particle nucleation. Accordingly, DFM measurements show a region of decreased intensity of the scattered light near the electrode surface when sufficiently high potentials are applied, as highlighted in orange in Figure 4D–F. In this region, Ag^+^ ions present in solution can adsorb on the AgCl layer, providing the enhancement of the Raman signal.^23^ This region is also visible in Figure S3 in the SI and in Video S2. Both show the oxidation of the silver microwire at a higher magnification, evidencing the region close to the electrode where mainly dissolved Ag^+^ is present, as the nucleation of AgCl particles is hindered by the low local chloride concentrations. In addition, Ag^+^ ions can diffuse away from the electrode until higher concentrations of chloride are reached, leading again to the generation of AgCl nanoparticles. This time, however, they are formed in solution. Thus, high intensities of scattered light were recorded for a long distance from the wire (≈60 μm), demonstrating the evolution of the diffusion layer.

Additionally, the evolution of the intensity at selected positions versus the applied potential provides further information on the AgCl generation (Figure 5, only the R component is shown; similar results were obtained for G and B components, data not shown). Two different positions were selected to follow the process. The first one was placed on the edge of the wire (red solid line in Figure 5) and the second one was placed close to the electrode (∼3 μm; red dotted line in Figure 5). In the absence of chloride, no changes in the normalized R component intensity values are detected (Figure 5A, red lines), as was expected, whereas in the LSV (Figure 5A, black line) the oxidation of the Ag surface to Ag^+^ is observed as an increase of the current at potentials from −0.20 V onward. Conversely, two different behaviors result in a 5 mM KCl solution (Figure 5B, red lines) for the two different positions checked. First, a linear decrease of the signal intensity on the wire from −0.35 to −0.20 V is observed due to the generation of a thin layer of nonuniform AgCl particles. Close to the wire, this decrease is only observed from −0.35 to −0.30 V. When exceeding the potential of −0.20 V, a linear increase in the intensity is detected in the measurements carried out close to the wire. This is related to the generation of AgCl particles in solution, while on the wire the increase is much less significant. These changes are consistent with the electrochemical response (Figure 5B, black line) in which a first oxidation peak is observed at around −0.38 V related to the oxidation of Ag to form the thin layer of AgCl particles, while at potentials above −0.20 V the generation of Ag^+^ is responsible for the current increase, as was previously discussed.

**Figure 6:**
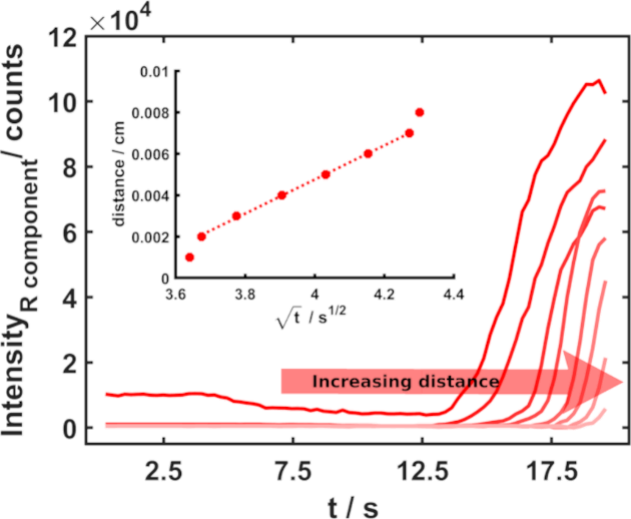
Evolution of the R component intensity with the applied potential in different positions, with distance from the wire increasing from left to right (from 10 to 80 μm, taking as a reference the edge of the wire). The inset shows the relationship between the distance chosen and the square root of the time for the onset potential. A good linear relationship is shown from 20 to 70 μm, *R*^2^ = 0.999.

Finally, when the amount of KCl is increased up to 50 mM, a remarkable difference is observed in the optical response between the wire (Figure 5C, solid red line) and a position close to the wire (Figure 5C, dotted red line). While on the wire a decrease of the normalized intensity by almost 90% is observed due to the generation of a thicker layer on the wire that decreases the scattered light, the intensity increases drastically in a position close to the wire due to the growth of this thick AgCl layer. The current response (Figure 5C, black line) shows a higher current peak around −0.32 V related to a higher AgCl formation on the surface of the wire, where the subsequent release of Ag^+^ was partially suppressed and each Ag atom that underwent oxidation contributed to the AgCl layer generation. The spatial evolution of the formation of AgCl particles in solution (due to Ag^+^ ions diffusing from the wire prior to AgCl particle nucleation) can be visualized by analyzing the evolution of the R component intensity versus the applied potential at different positions from the wire. Figure 6 shows the plot of the variation of intensity of the R component of the DFM images for positions from 10 to 80 μm from the edge of the wire. A more detailed description about this data analysis can be found in Figure S2B in the SI.

A decrease in the signal intensity is observed, indicating a lower generation of particles far away from the electrode surface. The inset shows the relation between the distance from the wire and the square root of time related to the Ag^+^ detection as AgCl bright particles. This time was obtained from the onset of the R component signal during the LSV at a fixed distance. From this relationship, it can be concluded that the process is controlled by different mechanisms. In the position closest to the wire, the process is probably controlled by the diffusion of Cl^–^ from the bulk solution, which is depleted during the generation of the layer of AgCl particles.^38,44^ After some distance/time, the process is mainly controlled by diffusion of silver ions from the wire surface^38,44^ (as is evidenced in the good linear relation between the distance from the wire and the square root of time, *R*^2^ = 0.999). For longer distances/times (higher than 70 μm), effects other than diffusion, such as, for example, convection, interfere in the particle generation.

### Particle Characterization

**Figure 7:**
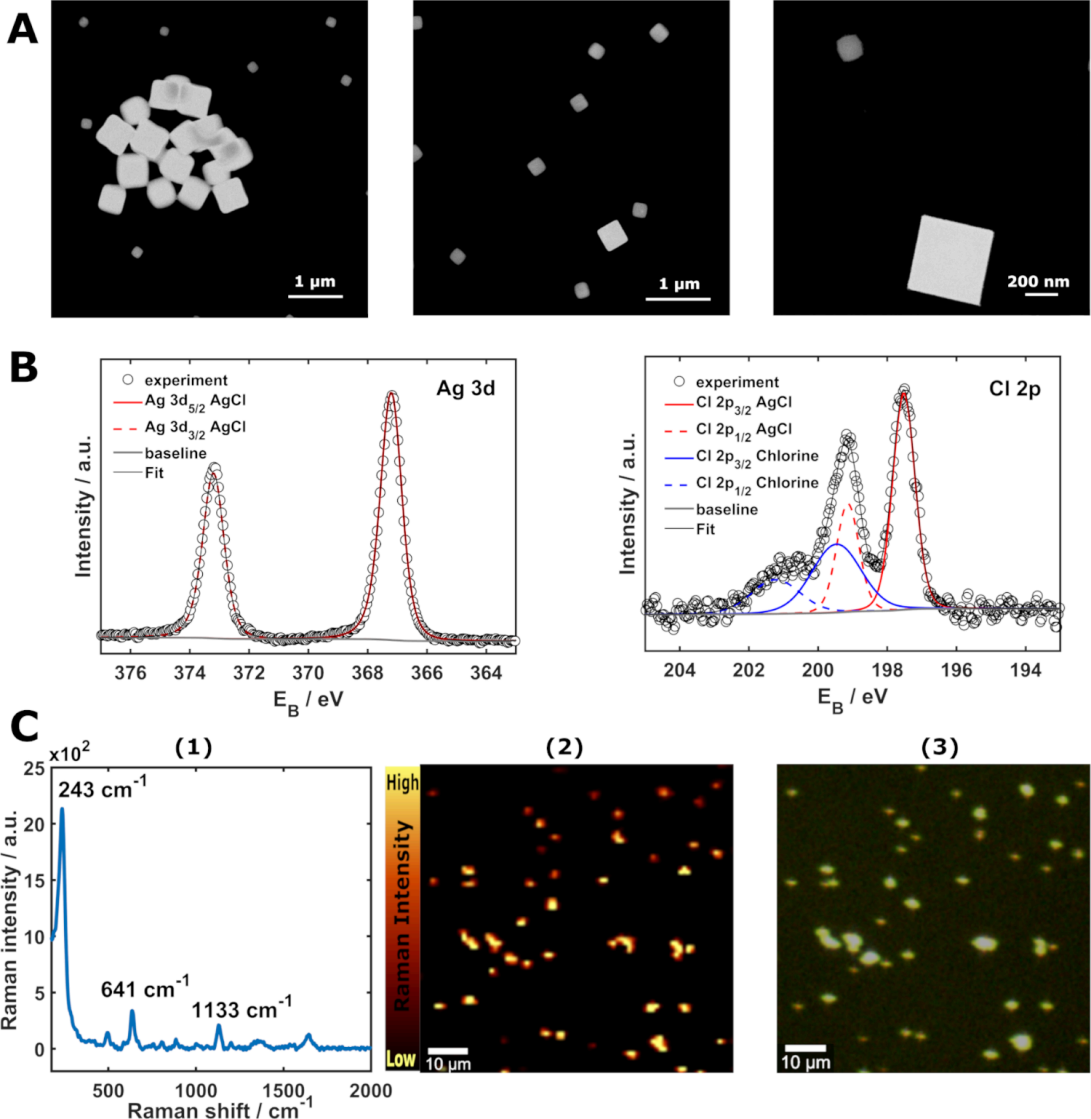
(A) STEM images, (B) XPS, and (C) Raman microscopy characterization of the electrogenerated nanoparticles. Raman spectra were obtained using a solution containing 140 μM uric acid, 10 mM Ag^+^, and 0.1 M HClO_4_. The spectrum shown in C(1) corresponds to the mean of the Raman mapping in C(2), while the corresponding optical image can be seen in C(3).

The electrogenerated AgCl particles were characterized by STEM, XPS and Raman microscopy, allowing us to obtain the complementary information displayed in Figure 7. The particles were generated following the experimental conditions described in Figure 2 and were coated onto a TEM grid, an ITO slide, and a glass slide for STEM, XPS, and Raman characterization, respectively. The samples were thoroughly rinsed with water and dried under nitrogen atmosphere prior to characterization; more information about sample preparation can be found in Figure S1 in the SI.

First, Figure 7A shows representative images of cubic particles formed by this electrochemical route, in agreement with previous studies.^19,24,46^ The images reveal a high dispersion in size, but two main populations can be easily distinguished: larger particles ranged between edge lengths of 400 and 500 nm and smaller ones ranged between edge lengths of 80 and 200 nm. Determination of the elemental composition of these particles by energy-dispersive X-ray spectroscopy (EDX) did not succeed, since the associated exposure to the electron beam led to the reduction of AgCl and the formation of metallic Ag nanoparticles on top of these cubes, as can be seen in Figure S4 in the SI. Therefore, the chemical composition of these particles was identified by XPS analysis (Figure 7 B).

The obtained XPS spectra are depicted in Figure 7 B. They show two peaks at 366.3 and 373.1 eV, corresponding to the binding energies for Ag 3d_5/2_ and Ag 3d_3/2_, respectively. These are related to Ag in AgCl.^47−49^ Two additional peaks recorded at 197.58 and 199.1 eV are associated with the binding energies for Cl 2p_3/2_ and Cl 2p_1/2_ in AgCl, respectively.^47−49^ The XPS results confirm the Ag_1_Cl_0.84_ composition of these nanocubes, which is near the expected stochiometric ratio, and suggest that Ag adsorbs on the AgCl cubes, which would explain a higher proportion of Ag (details of the peak fitting provided in Table S1 in the SI). The observation of a peak at 198.6 eV hints at the presence of residual chlorine, aligning with findings by Copperthwaite and colleagues, who postulated the existence of a stable surface hole species in irradiated AgCl, supported by a ≈2 eV increase in Cl 2p binding energy compared to the standard lattice Cl^–^. This increase implies the presence of a species with an electronic charge close to zero, which could be assigned as the self-trapped hole Cl_2_^–^. Alternative interpretations suggest that the second doublet is linked to the formation of neutral chlorine.^50^ The survey spectrum (Figure S5 in the SI) shows no additional elements beyond Ag and Cl, except for the contribution from the substrate.

Finally, we examined these particles with Raman microscopy following the strategy proposed in a recent work.^23^ The particles themselves do not cause an enhancement of the Raman signal when a test molecule is present in the solution, and only the Raman band related to AgCl can be observed (data not shown). However, when the test molecule (uric acid) was added in the presence of Ag^+^ cations, the Raman spectrum of the test molecule is clear, as can be seen in Figure 7C(1). The comparison between the Raman mapping, Figure 7C(2), and the optical image, Figure 7C(3), of the particles under these conditions clearly shows that the spectrum of uric acid is present only where the nanoparticles are placed.

In conclusion, STEM images confirm the generation of particles in solution, as observed by DFM. They also provide information about the size and shape of these particles. Meanwhile, XPS analysis provides further evidence of the chemical composition, confirming the generation of AgCl particles and supporting the observations made during our operando experiments. In addition, an enhancement of the Raman signal can be obtained using these particles in the presence of Ag^+^ ions. This is in agreement with previous studies, indicating that this phenomenon is cation-mediated by Ag^+^, potentially linking the molecules and the solid particles.^23^ We thus reveal that these Ag^+^ modified AgCl nanocrystals can be used as a SOERS-active material in a similar way that nanoparticles are used for SERS. Accordingly, we suggest that due to the composition of these crystals, charge transfer between the Ag adsorbed on the AgCl cubes and the AgCl may modify the electronic and optical properties of these AgCl particles. This promotes a charge transfer between these structures and the adsorbed molecules, enabling the nanocrystals to strongly enhance Raman signals.

## Conclusions

In this work, Ag microwires have been used to investigate the electrolytic conditions to generate SOERS active substrates. This new setup allows us to follow the whole process *operando* with DFM, providing dynamic information about it in a simple way.

The analysis of the RGB components of the DFM video images recorded during the electrochemical experiments allows us to evaluate the effect of the Cl^–^ concentration, showing an *operando* picture of the processes taking place on the surface of a silver electrode and in the solution adjacent to it. These results have implications for both EC-SOERS and EC-SERS substrate generation. On the one hand, high Cl^–^ concentrations generate a thick AgCl layer on the silver microwire, which allows the accumulation of Ag^+^ and generates highly active SERS substrates by AgCl reduction. On the other hand, the EC-SOERS phenomenon is only observed at low Cl^–^ concentrations, in which electrochemically released Ag^+^ ions can adsorb on the nonuniform AgCl nanocrystals layer, enhancing the Raman signal, while the rest of Ag^+^ ions diffuse away from the electrode surface until reaching the Cl^–^ from the bulk, generating AgCl nanocrystals in the solution.

STEM images demonstrate that these ions generate cubic particles, and XPS analysis confirms the AgCl composition. These crystals are also able to enhance the Raman signal when Ag^+^ is added to the solution, which was confirmed by Raman microscopy. Our results clearly reveal that the SOERS effect is enabled by Ag^+^ ion-modified AgCl particles and their specific properties. Therefore, the Cl^–^ concentration is the key factor, enabling a balance between the single generation of Ag^+^ ions and the single generation of the AgCl precipitate.

Providing both understanding of the mechanism of SOERS substrate formation and a demonstration of micrometer-sized SOERS substrates (microwires or active nanocrystals), our results open new doors to the generation and design of SOERS substrates and experiments, allowing the move from large sensors to miniaturized applications, such as microneedle-based sensors for healthcare applications or flexible microsensors for environmental control.

## Supporting Information Available

Electrochemical setup and sample preparation, description of data analysis, DMF images of the silver wire oxidation at higher magnification, effect of the exposure time in STEM images, and XPS survey and peak fitting details (PDF)
Oxidation of a Ag microwire during LSV from −0.45 (OCP) to −0.05 V vs PtIr in 0.1 M HClO_4_ and 5 mM KCl at a scan rate of 0.02 V·s^–1^ (AVI)
Higher-magnification version of Video S1 (AVI)

## Supplementary Material

ac4c05942_si_001.pdf

ac4c05942_si_002.avi

ac4c05942_si_003.avi
