# Supplementary material for: The Role of Chloride in Raman Signal Enhancement by Electrochemical Silver Oxidation Revealed by Dark Field Microscopy
Source: Anal Chem. 2025 Apr 1;97(14):7772–80. doi: 10.1021/acs.analchem.4c05942 (PMC12004349; doi:10.1021/acs.analchem.4c05942)
Supplement: Supplementary file 1 — ac4c05942_si_001.pdf [file ac4c05942_si_001.pdf]

Supporting information

The Role of Chloride in Raman Signal Enhancement by Electrochemical Silver Oxidation Revealed by Dark Field Microscopy

Sheila Hernandez*[1,2], Kevin Wonner[1], Pouya Hosseini[3], Paolo Cignoni[1], Aranzazu Heras[2], Alvaro Colina[2], Kristina Tschulik*[1,3].

[1] Chair of Analytical Chemistry II, Faculty of Chemistry and Biochemistry, Ruhr University Bochum, Bochum 44801, Germany

[2] Department of Chemistry, Universidad de Burgos, Pza. Misael Bañuelos s/n, E-09001 Burgos, Spain

[3]Max-Planck-Institut fürNachhaltige Materialien GmbH, Max-Planck-Straße 1, 40237 Düsseldorf, Germany

Contents

S1. Electrochemical setup and sample preparation. 2

S2. Description about data analysis. 3

A. Representation of how intensity changes are plotted along the space in Fig. 4 3

B. Representation of how intensity vs time plots shown in Fig. 6 were derived. 3

S3. DFM images of the silver wire oxidation at higher magnification. 4

S4. Effect of the exposure time in STEM images. 5

S5. XPS survey 6

S6. XPS peak fitting details. 7

S7. DFM Video description. 8

S8. REFERENCES 9

S1. Electrochemical setup and sample preparation.

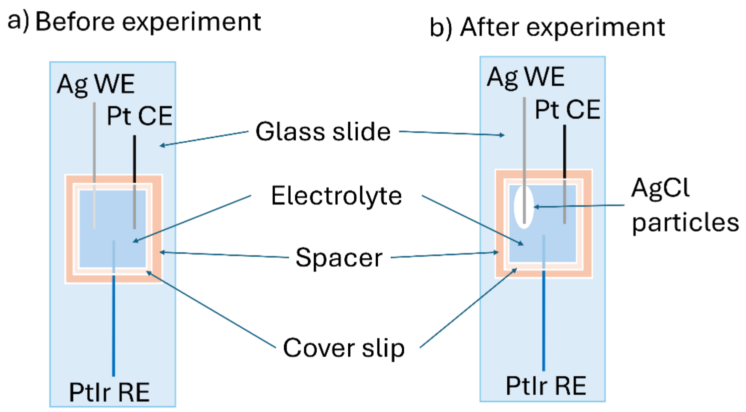

Figure. S1. Scheme of the DFM/Raman electrochemical cell before (a) and after (b) the experiments (top view). A new cell[1] with new wires and fresh electrolyte solution was used for each experiment. The white part shown in Figure S1b represents the synthesised AgCl particles. This scheme provides a top view of the cell after the experiment. For STEM characterization a TEM grid was place below the Ag wire (without being in contact), so that some of the particles generated in front of the Ag wire were collected on the grid. In the case of XPS analysis, an ITO slide was used instead of the glass slide, following the same design, and adding a 3D printed support for the ITO to avoid electrical contact between the ITO and the wire electrodes. For Raman experiments the cell was used as shown in Figure S1a for the in-situ experiments (Figure 1), and the wires were removed and the cell rinsed with water to register the Raman mapping of the particles (Figure 7C). It should be noted that the particles are slightly adhered to the surface. Hence, the solution was removed/replaced by rinsing solution after the experiments, while keeping the particles in the same position. For cleaning aprox. 2 mL of ultrapure water was used to rinse the particles in an immersing fashion, instead of flushing the water across the particles, which would detach the particles. This cleaning step was done for few seconds each and repeated 3 times to prevent nanoparticle dissolution.

S2. Description about data analysis.

A. Representation of how intensity changes are plotted along the space in Fig. 4.

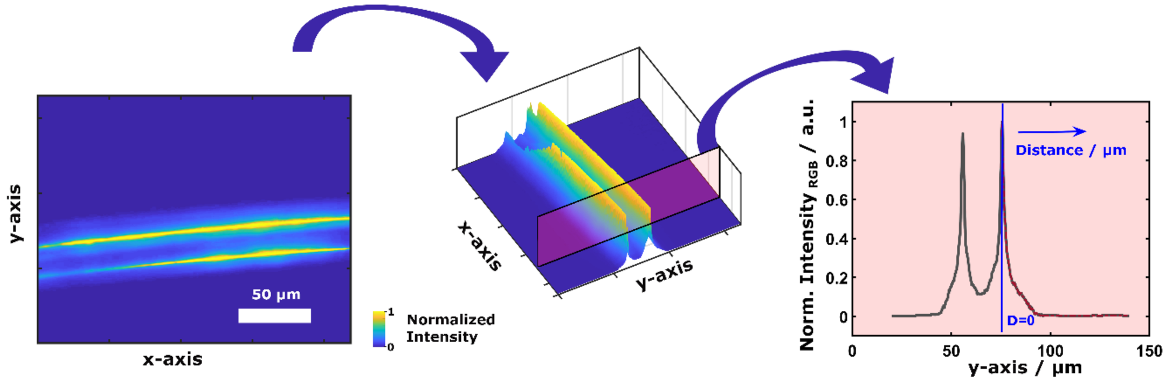

Fig. S2-A. Representation of the data treatment shown in Fig.4. Contour plot (left) and 3D representation of the normalized intensity (middle) of DFM images shown in Fig. 3 in the main text The line scan across the wire (right) is used for the distance reference in Fig.4 in the main text, considering the blue line as D=0;

B. Representation of how intensity vs time plots shown in Fig. 6 were derived.

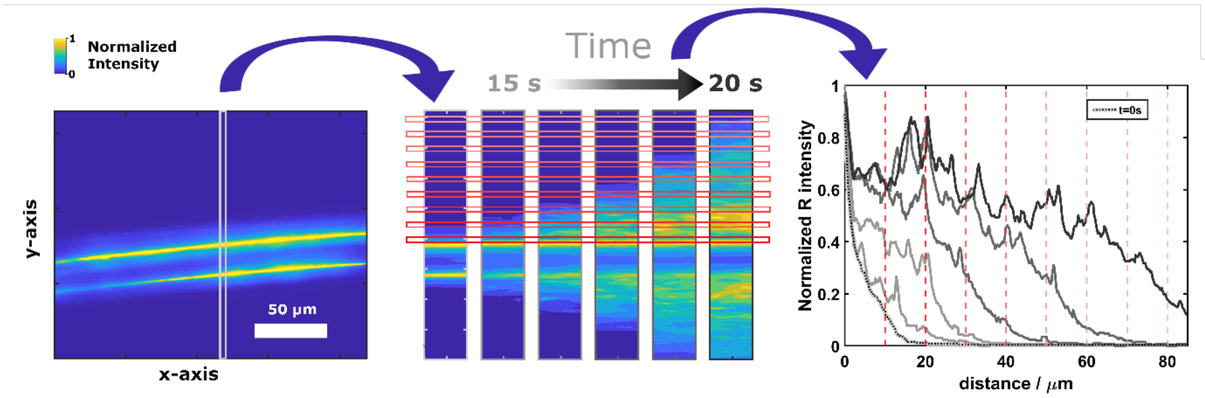

Fig. S2-B. Representation of the data treatment shown in Fig.6. Contour plot (left) and a section of the contour plot of 4 μm length (x-axis) at different times during the experiment (middle). The right panel shows the evolution of the intensity at different times (grey and black lines). Red lines represent the different positions considered in Fig. 6. Time is related to the applied LSV, which starts at -0.45 V up to -0.05 V vs PtIr, at a scan rate of 0.02 V·s^{-1}. Therefore, tracking the evolution of the intensity at different positions from the wire, and considering that the changes of the intensity are related to the generation of AgCl, allows us to analyse the diffusion of Ag^+ cations.

S3. DFM images of the silver wire oxidation at higher magnification.

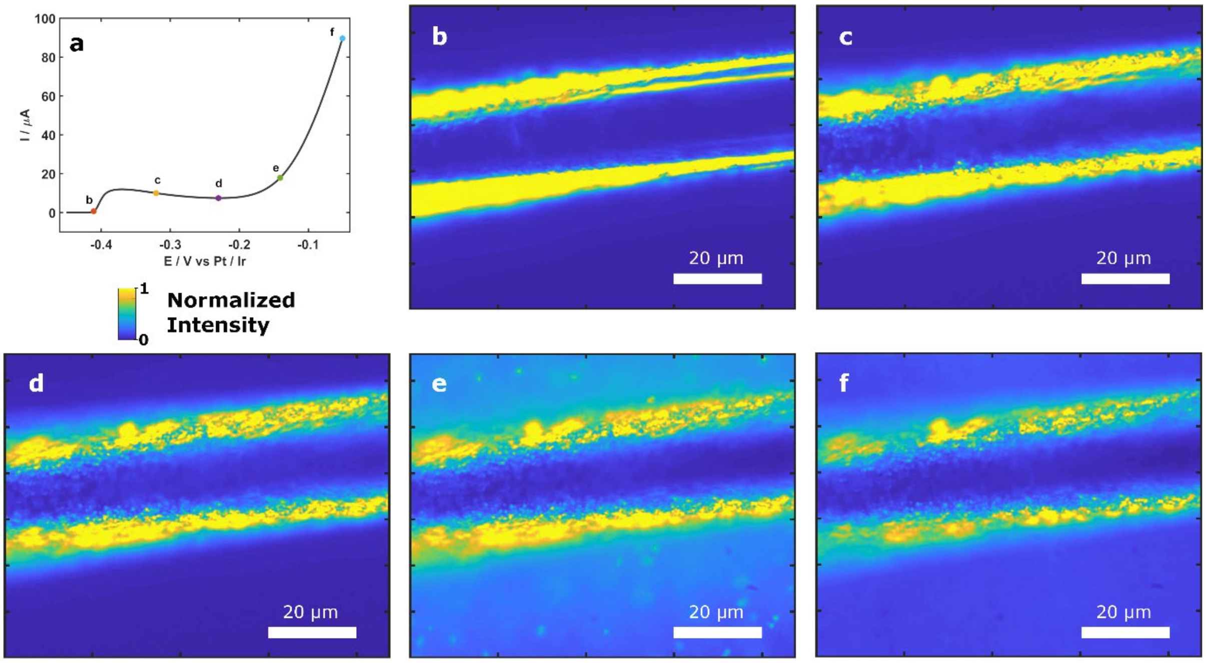

Fig. S3. LSV at a Ag micro-wire in 0.1 M HClO4 and 5 mM KCl (a) and normalized intensity of the R component of DFM images (b, c, d, e, f) obtained at different potentials (labelled in Fig. S3a), during the LSV. DFM images were collected with an oil immersion 100x objective. These images are extracted from video S2.

S4. Effect of the exposure time in STEM images.

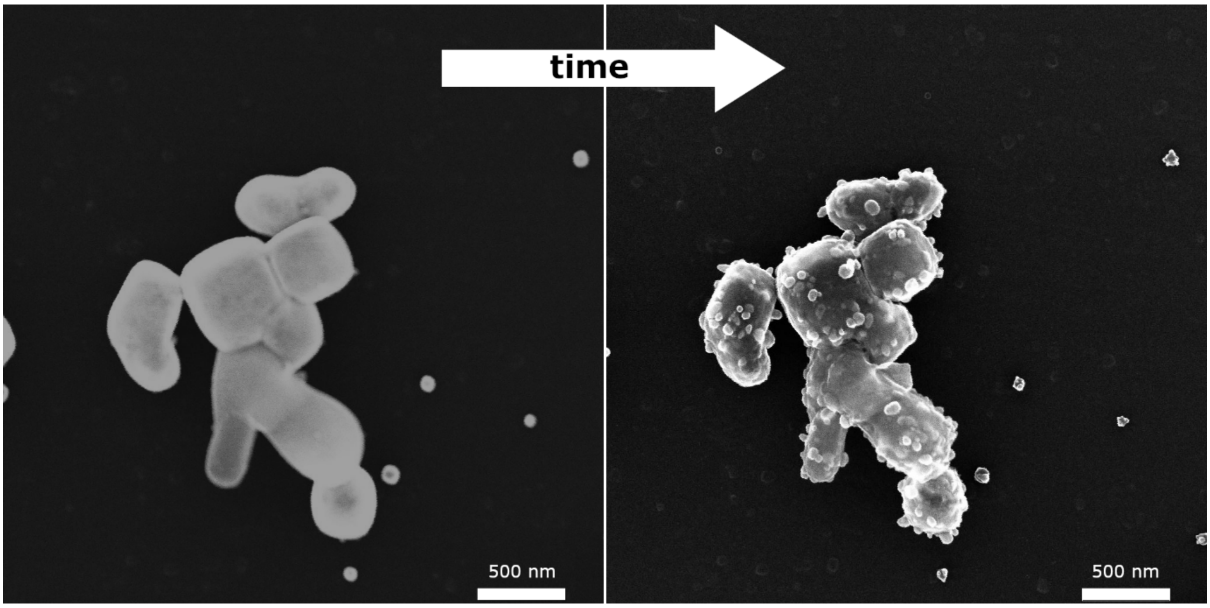

Fig. S4. Effect of the electron irradiation on AgCl cubes evidenced by STEM using the secondary electron signal. The extensive exposure to the electron beam led to the evolution of AgCl cubes with time, generating metallic Ag nanoparticles on top of these cubes. This (undesired) beam effect induces sample alteration and limits the analysis of the formed nanocrystals and their elemental composition by EDX.

S5. XPS survey

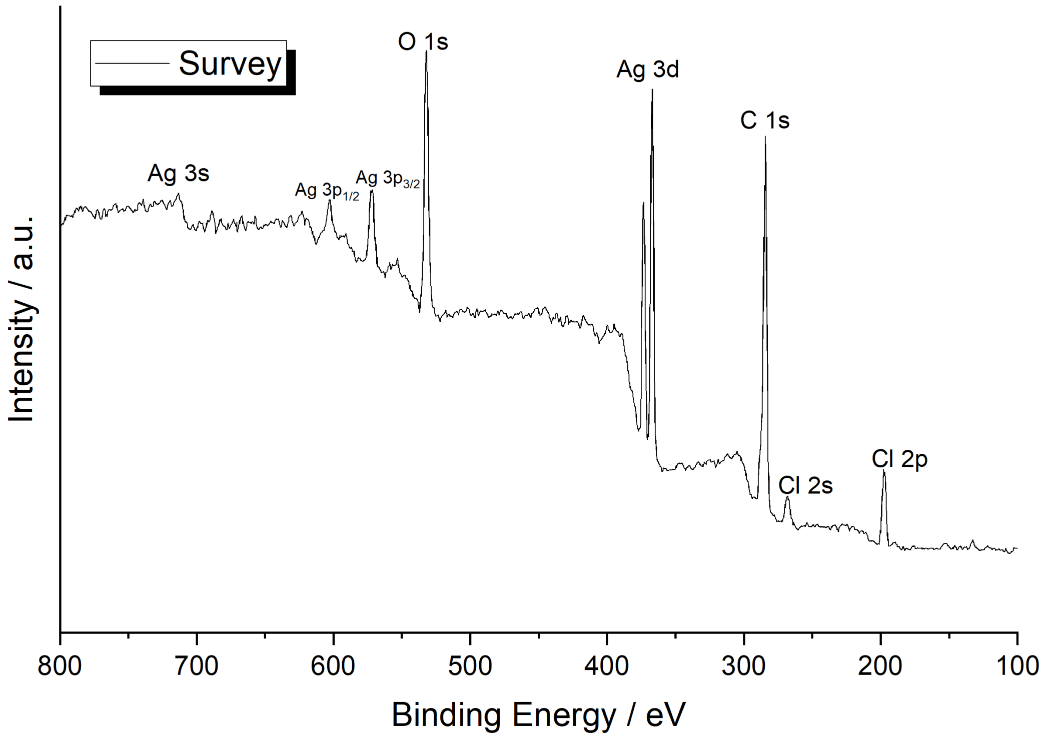

Fig. S5. XPS survey spectrum of the electrogenerated nanoparticles. The spectrum shows no additional elements beyond Ag and Cl, except for the typical carbon and oxygen peaks, which originate from the substrate from sample preparation.

S6. XPS peak fitting details.

Table S1. Ag 3d and Cl 2p spectral fitting parameters (BE.: Binding energy, FWHM: full width at half-maximum, and L.Sh.: line shape).

	Peaks	BE. (eV)	FWHM	L.Sh.	Area	%Area
Ag	$3d_{5/2}$ AgCl	367.2	0.84	LA(1.61,1.8,80)	211778.60	60
	$3d_{3/2}$ AgCl	273.2	0.84	LA(1.61,1.8,80)	141185.73	40
Cl	$2p_{3/2}$ AgCl	197.5	0.77	LA(1.53,243)	25078.72	39
	$2p_{1/2}$ AgCl	199.1	0.77	LA(1.53,243)	12539.36	20
	$2p_{3/2}$ Chlorine	199.4	1.68	LA(1.53,243)	17320.61	27
	$2p_{1/2}$ Chlorine	201.2	1.68	LA(1.53,243)	8660.31	14

S7. DFM Video description.

Video 1. The video shows the oxidation of a Ag micro-wire during a LSV from -0.45 V (OCP) to -0.05 V vs PtIr, in 0.1 M $HClO_4$ and 5 mM KCl and at a scan rate of 0.02 $V \cdot s^{-1}$. It was recorded with a CCD camera with an integration time of 250 ms, using an air 40x magnification objective. The corresponding LSV curve is displayed in Figure 2 in the main text.

Video 2. The same experiment as presented in VideoS1 has been performed with a higher magnification, in this case an oil immersion 100x objective was used, while other experimental conditions were the same as described above. The correlated data (LSV and R component analysis) are shown in Figure S3.

S8. REFERENCES

(1) Wonner, K.; Evers, M. V.; Tschulik, K. Simultaneous Opto- and Spectro-Electrochemistry: Reactions of Individual Nanoparticles Uncovered by Dark-Field Microscopy. J. Am. Chem. Soc. 2018, 140 (40), 12658–12661. https://doi.org/10.1021/jacs.8b02367.
